# Supplementary material for: Amphibians and Reptiles of the Montagne des Français: An Update of the Distribution and Regional Endemicity
Source: Animals (Basel). 2023 Oct 29;13(21):3361. doi: 10.3390/ani13213361 (PMC10648909; doi:10.3390/ani13213361)
Supplement: Supplementary file 1 [file animals-13-03361-s001.zip › Figure S1-Photo of amphibians.pdf]

AMPHIBIAN SPECIES

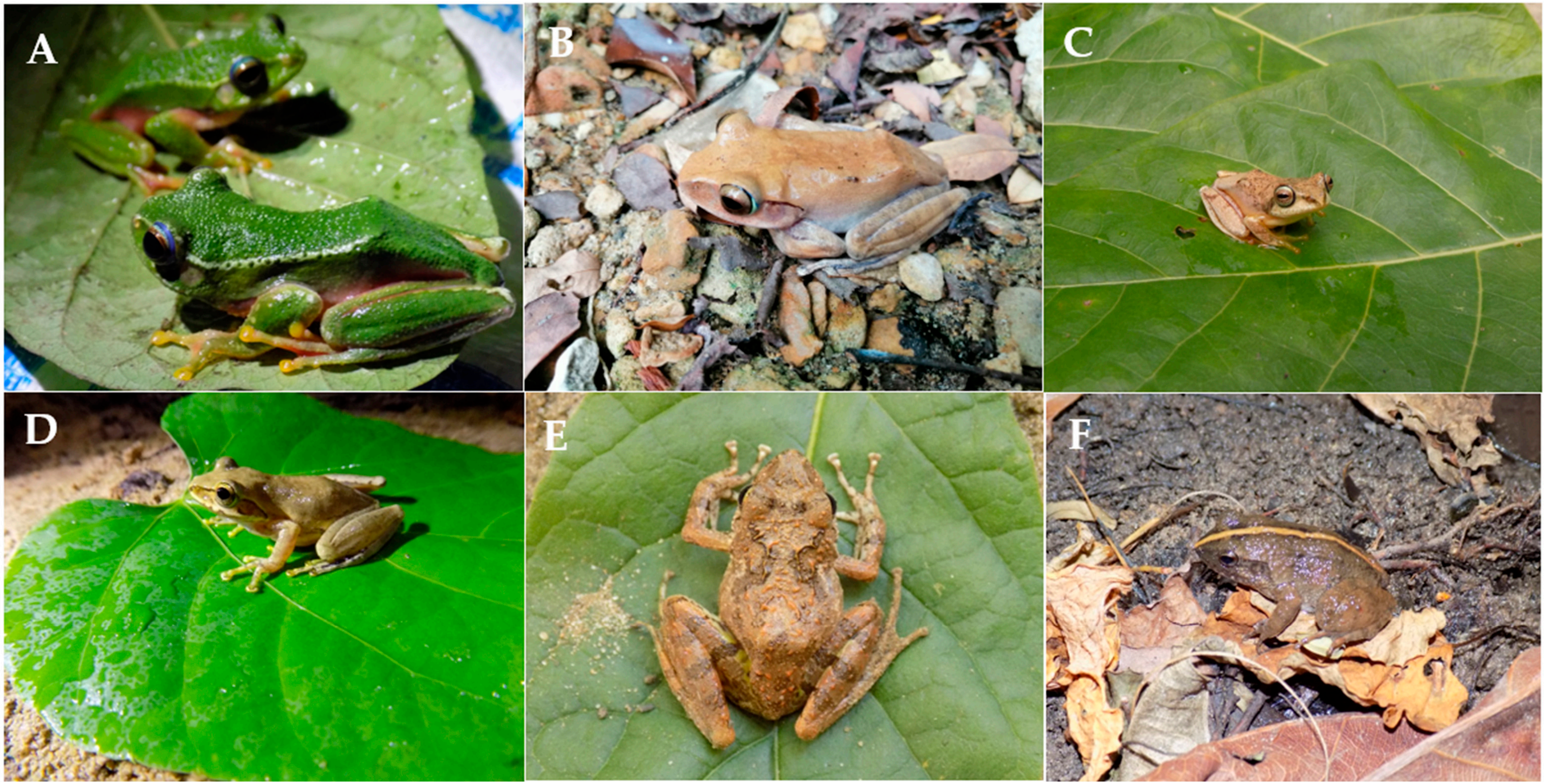

A : *Boophis* cf. *occidentalis* ; B : *Boophis* sp. ; C : *Boophis* cf. *marojezensis* ; D : *Boophis tephraeomystax* ; E : *Gephyromantis pseudoasper* ; F : *Mantidactylus ulcerosus*

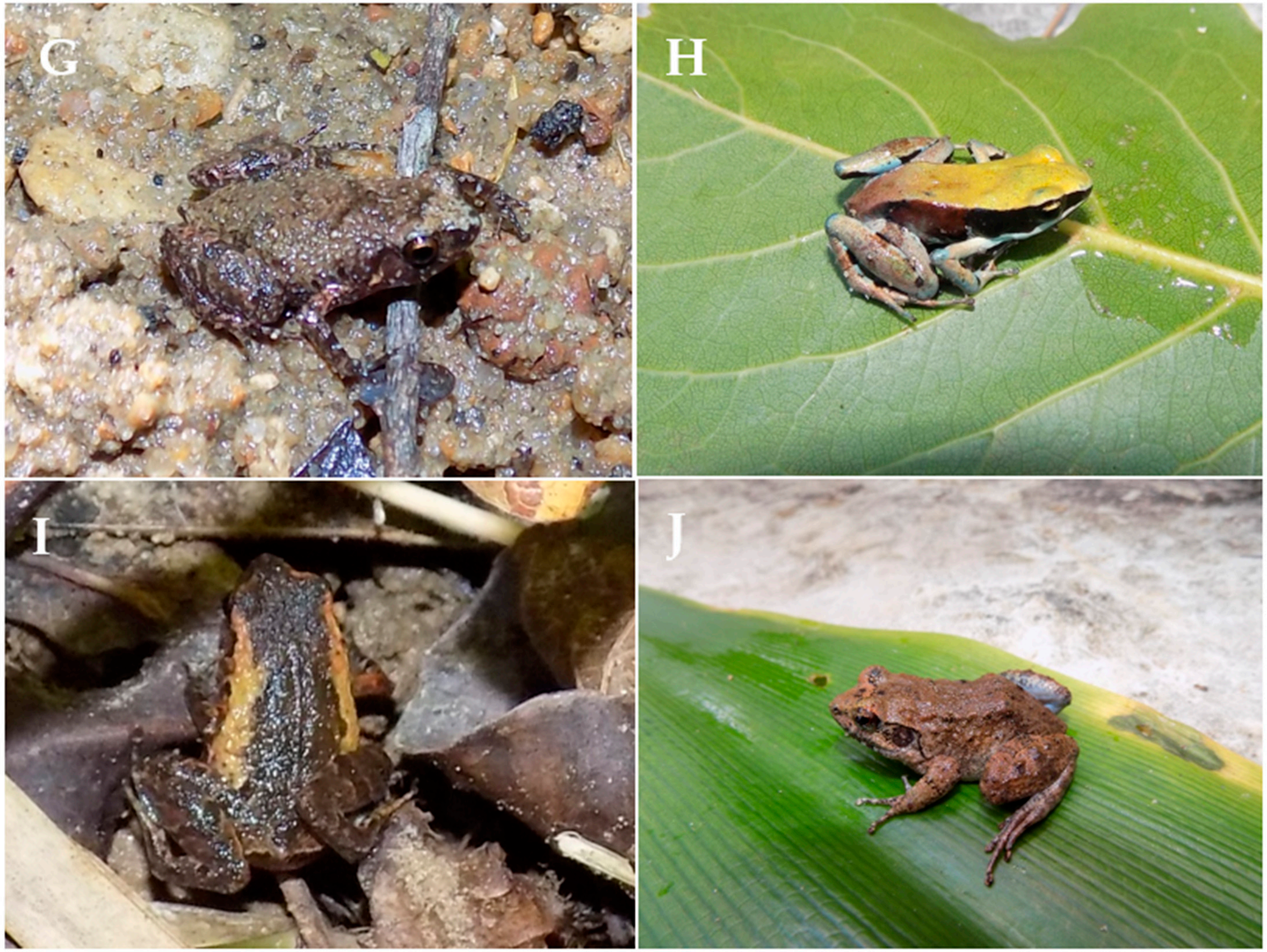

G : *Stumpffia roseifemoralis* ; H : *Mantella viridis* ; I : *Stumpffia* cf. *madagascariensis*; J : *Mantidactylus bellyi*
