# Supplementary material for: Amphibians and Reptiles of the Montagne des Français: An Update of the Distribution and Regional Endemicity
Source: Animals (Basel). 2023 Oct 29;13(21):3361. doi: 10.3390/ani13213361 (PMC10648909; doi:10.3390/ani13213361)
Supplement: Supplementary file 1 [file animals-13-03361-s001.zip › Figure S2-Photo of reptiles.pdf]

REPTILE SPECIES

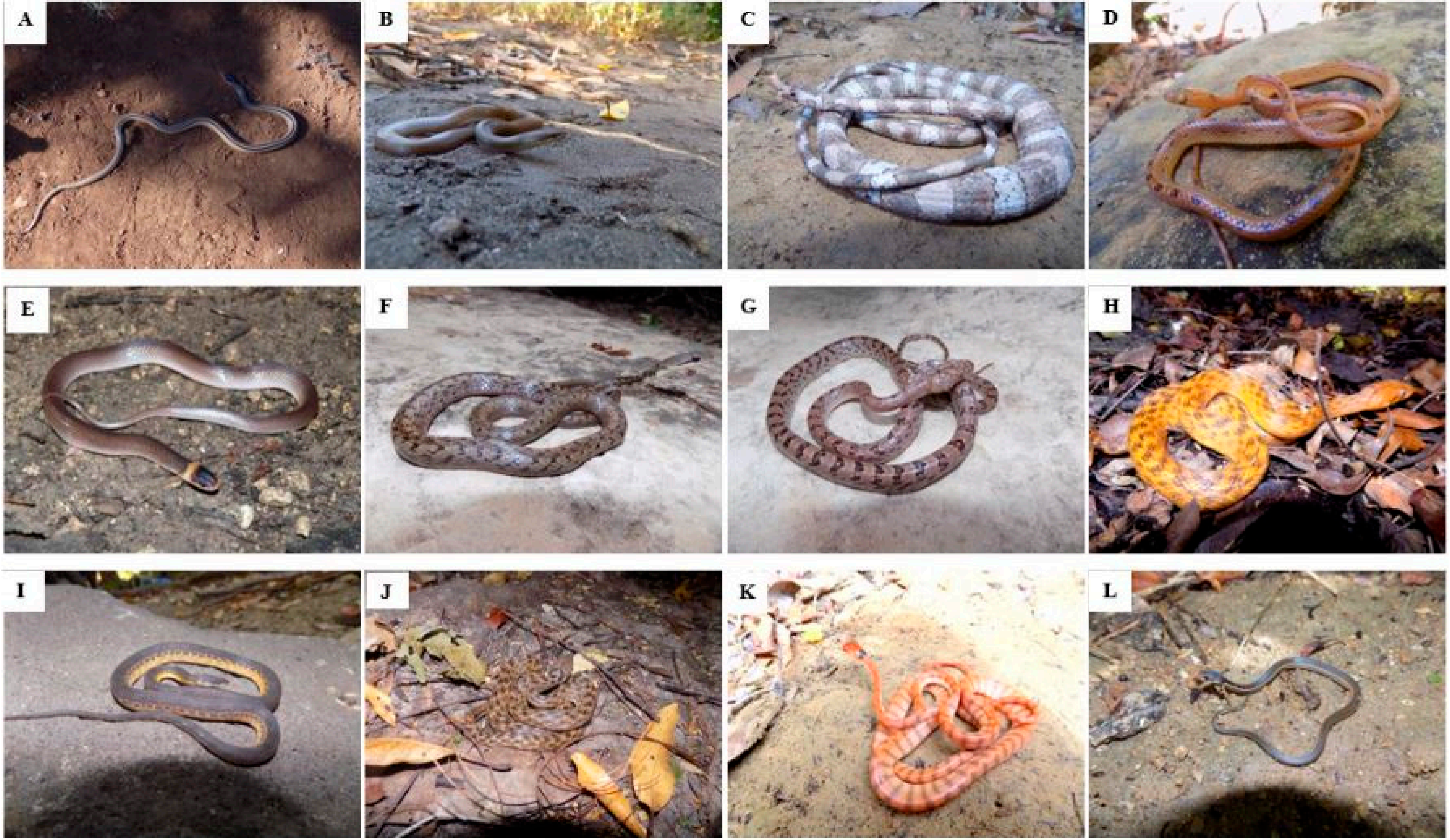

A) Dromicodryas quadrilineatus, B) Liophidium torquatum, C) Langaha pseudoalluaudi, D) Lycadryas inopinae, E) Heteroliodon fohy , F) Thamnosophis martae, G) Lycodryas pseudogranuliceps, H) Madagascarophis fuchsi, I) Alluaudina belly, J) Madagascarophis colubrinus, K) Physalixella sp., L) Thamnosophis stumpffi.

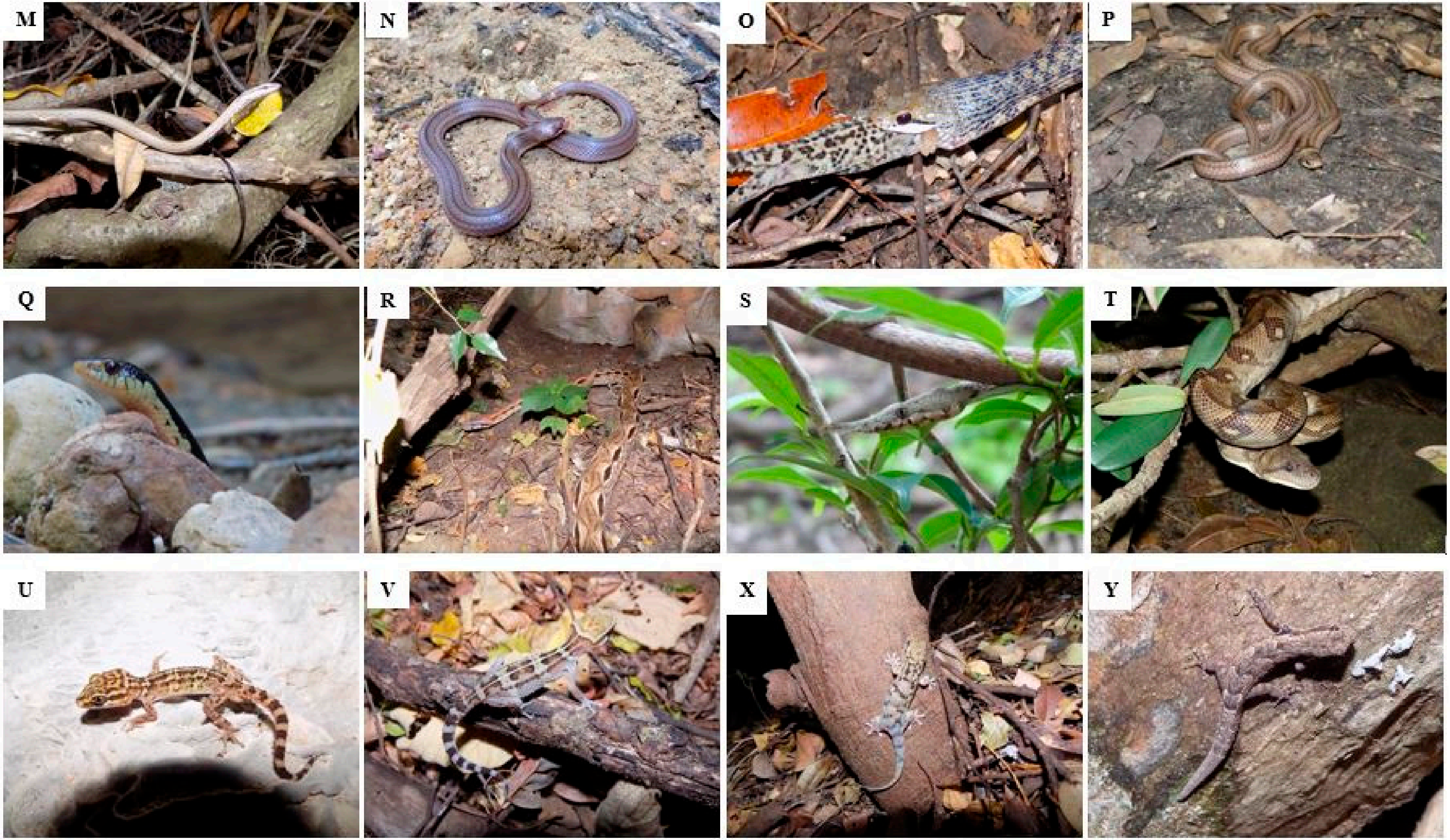

M) Mimophis occutlus, N) Pseudoxyrhopus quinquelineatus, O)Thamnosophis martae end Paroedura stumpffi , P)Luiphidium therezieni, Q)Leioheterodon madagascariensis ,R) Acrantophis madagascariensis, S) Langaha madagascariensis, T) Sanzinia volotany U) Paroedura lohatsara, V)Paroedura stumpffi, X)Geckolepis maculata, Y)Lygodactylus heterurus

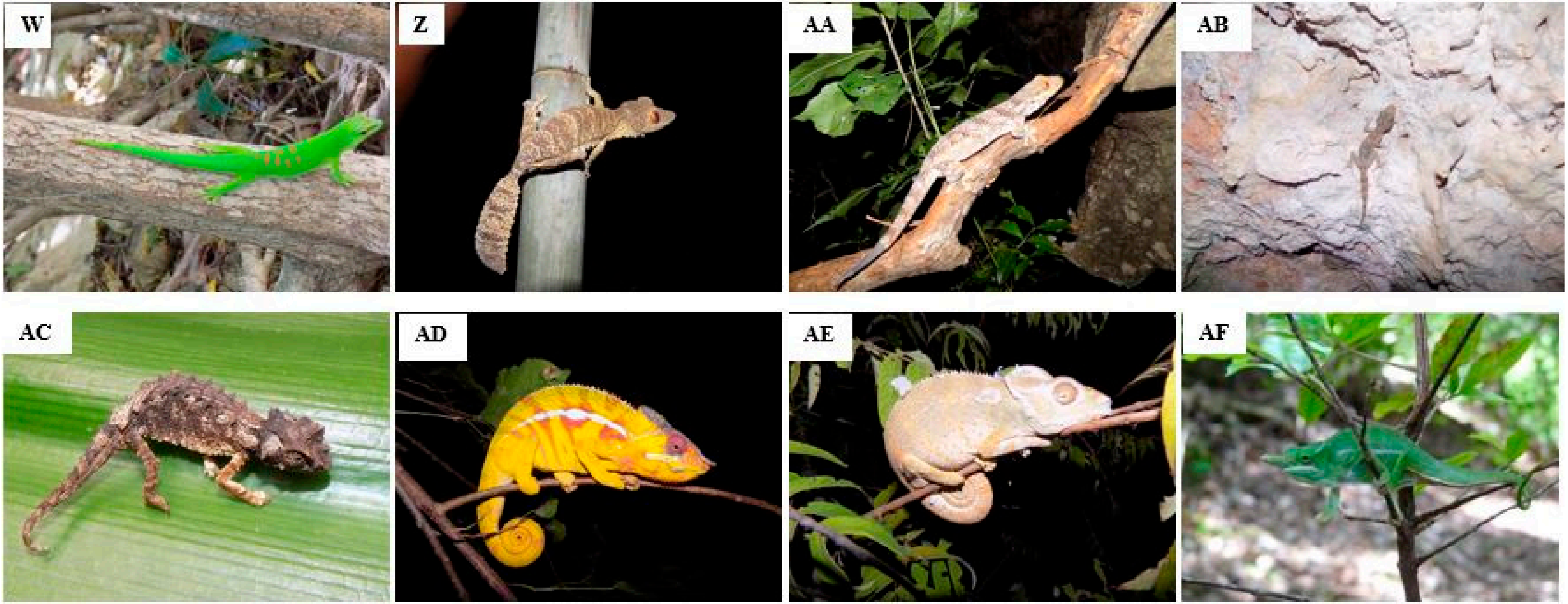

W) Phelsume grandis, Z) Uroplatus henkeli, AA) Blaesodactylus boevini, AB) Paroedura hordiesi, AC) Brookesia ebenaui, AD Furcifer pardalis, AE) Furcifer oustaleti, AE) Furcifer petteri
